# The Protective Role of Social Support Against Depression, Anxiety, and Stress in Physiotherapy Students

**DOI:** 10.3390/ijerph23010082

**Published:** 2026-01-07

**Authors:** Latifah Alenezi, Maath Alhaddad, Shareefah Almutairi, Fareedah Almohri

**Affiliations:** Physical Therapy Department, Faculty of Allied Health Sciences, Kuwait University, P.O. Box 31470, Sulaibekhat, Kuwait City 90805, Kuwait; maath.alhaddad@ku.edu.kw (M.A.); shareefah.almutairi@ku.edu.kw (S.A.); fareda.almahri@ku.edu.kw (F.A.)

**Keywords:** DASS-21, depression, anxiety, stress, social support, physiotherapy students

## Abstract

**Background:** Physiotherapy University students frequently experience psychological challenges such as depression, anxiety, and stress. These issues are shaped by various individual, health-related, and social factors. Understanding these influences is essential for identifying vulnerable subgroups and guiding effective support strategies. **Aim and Objectives:** This study aims to assess the prevalence and severity of depression, anxiety, and stress among physiotherapy students. Specific objectives include (1) analyzing mental health variations across demographic variables such as gender, nationality, marital status, and year of study; (2) evaluating the impact of physical and mental health conditions on psychological distress; and (3) investigating the protective role of perceived social support from family, friends, and faculty. **Methods:** A cross-sectional study was conducted among 282 physiotherapy students at Kuwait University, focusing on students from the Physical Therapy Department in the College of Allied Health Sciences, of whom 89% were aged 18–22 years and 10% were aged 23–27 years. Participants were selected through purposive and convenience sampling, including second-, third-, and fourth-year students. Data were collected using the DASS-21 scale along with demographic, health, and social support information. Descriptive statistics and ANOVA tests were used to analyze associations between psychological outcomes and the studied variables. **Results:** Anxiety was the most prevalent condition, with 39.4% of students reporting extremely severe levels. Depression and stress affected 14.2% and 11.3% of students at the same severity level, respectively. Gender differences were examined among the participants (259 females and 23 males), with female students showing significantly higher stress levels than males and second-year students reporting greater depression and stress compared to their senior peers. Students who received support from family and friends exhibited lower levels of psychological distress. **Conclusions:** This is the first study in Kuwait to examine mental health among physiotherapy students, revealing a high prevalence of depression, anxiety, and stress in this group. The findings highlight the unique academic and clinical pressures faced by physiotherapy students, including intensive coursework, frequent assessments, physically demanding clinical placements, close patient interaction, and the need to integrate theoretical knowledge with hands-on clinical practice, while also emphasizing the protective role of social support. These insights call for targeted mental health strategies and support systems within physiotherapy education to promote student well-being and foster a healthier learning environment.

## 1. Introduction 

Social support refers to the perception and reality of being cared for, having assistance available, and belonging to a supportive network [1,2]. It includes emotional, instrumental, informational, and appraisal support [3]. Evidence consistently shows its protective role against psychological distress among university students, with strong social networks linked to reduced depression, anxiety, and stress [4,5]. In physiotherapy education, social support may buffer the impact of academic and clinical stressors, making it critical for student well-being [6].

Mental health challenges among university students have become a global concern, particularly in demanding programs such as physiotherapy [7,8]. Physiotherapy students face unique stressors beyond typical academic pressures: they must master complex theoretical content, adapt to physically demanding clinical placements, and manage patient care responsibilities in diverse healthcare settings [9,10]. These stressors, combined with personal and societal expectations, can lead to depression, anxiety, and stress [11,12,13,14,15,16].

Previous studies report that physiotherapy students experience higher stress and burnout compared to peers in other disciplines [10,17]. Stress is strongly associated with anxiety and depression, and early intervention is essential to prevent long-term consequences [12,18]. Clinical placements, while integral to training, often amplify psychological strain [18]. McConville et al. [19] emphasized the significant influence of academic stress on mental health among physiotherapy students, reinforcing the need for targeted support.

Globally, mental health issues among physiotherapy students are influenced by demographic and psychosocial factors [20,21]. However, little is known about these challenges in Kuwait, where physiotherapy education spans four years, with clinical rotations beginning in the third year and intensifying in the fourth. This structure may create distinct stress patterns: second-year students face academic transition, third-year students adapt to clinical exposure, and fourth-year students manage career pressures.

Despite growing research, gaps remain in understanding mental health among physiotherapy students in Kuwait and the role of social support in mitigating distress. Recent evidence from Kuwait University demonstrated that a culturally tailored stress management program significantly reduced stress and depression among health science students, highlighting the importance of structured interventions in this context [22]. This underscores the need for complementary strategies, such as social support, to further enhance student well-being. This study addresses these gaps by (1) assessing the prevalence and severity of depression, anxiety, and stress using the DASS-21 scale; (2) examining variations by gender and year of study; and (3) evaluating the protective role of perceived social support from family, friends, and faculty. By doing so, the study seeks to provide insights that can inform the development of effective support systems within the College of Allied Health Sciences, ultimately enhancing student well-being and academic performance, as advocated by Alotaibi et al. [23], who emphasized the need for counseling and mental health services for university students.

**Hypotheses:** 

**H1:** Based on previous studies demonstrating the buffering effect of social support on psychological distress among university students [4,5], we hypothesize that higher perceived social support from family, friends, and faculty will be associated with lower levels of depression, anxiety, and stress among physiotherapy students.

**H2:** Consistent with research indicating that students in earlier academic years experience greater emotional vulnerability during transitional phases [24], we hypothesize that second-year students will report higher psychological distress compared to third- and fourth-year students.

**H3:** In line with evidence that female students are more likely to experience anxiety and stress than male students in healthcare programs [25,26], we hypothesize that female physiotherapy students will exhibit higher stress and anxiety levels than their male counterparts.

## 2. Materials and Methods

### 2.1. Study Design

This cross-sectional study was conducted at Kuwait University, targeting students from the Physical Therapy Department in the College of Allied Health Sciences. The study aimed to investigate the prevalence of depression, anxiety, and stress symptoms among undergraduate physiotherapy students and identify associated risk factors.

### 2.2. Sample

Participants were selected through a combination of purposive and convenient sampling, encompassing students in their second, third, and fourth years of study. The inclusion criteria for the study participants were (1) students from the Allied Health Sciences Faculty, specifically the Physical Therapy Department, (2) both male and female students, and (3) students in their second, third, and fourth years of study. The exclusion criteria included students from other faculties or departments at Kuwait University and first-year students. First-year students were excluded due to the low educational experience and lack of physical therapy courses in this academic year. Participants who met the inclusion criteria and agreed to participate in the study were considered approved study participants. The total number of physical therapy students in second, third and fourth years of study was 317, with 282 students agreeing to participate and complete the questionnaire. 

### 2.3. Data Collection

The local Institutional Review Board (IRB) approval was obtained from the Health Science Center (HSC) ethical committee at Kuwait University prior to the start of the data collection process (VDR/EC-26). Participants were contacted by research team members to explain the rationale and need for this study. Information about the study was shared through Microsoft Teams, WhatsApp groups, and QR codes distributed during in-person classes. Announcements were posted on relevant course channels, and virtual information sessions were held to explain the study’s objectives and procedures. During these sessions, students had the opportunity to ask questions and express their interest in participating. Participants’ anonymity and confidentiality were assured, and informed consent was obtained from all participants through an online consent form; QR codes linking to the online consent form and survey were posted on classroom, making it easy for students to scan and access the survey using their smartphones. The online scale was self-administered and sent to all students by email, MS Teams, WhatsApp, and social media platforms.

### 2.4. Instrumentation 

Two components were used for data collection:

**Demographic Data Sheet:** This included 11 items covering age, sex, nationality, marital status, major, year of study, medical condition, and sources of stress. It also incorporated three self-developed questions to assess perceived social support from family, friends, and faculty members. Responses to these items were recorded using a frequency-based scale: *Always*, *Frequently*, *Sometimes*, *Rarely*, and *Never*.
**Depression, Anxiety, and Stress Scale-21 (DASS-21):** This scale consists of three subscales: stress, anxiety, and depression. Each subscale has seven items, totaling 21 items. The scale assesses the frequency of symptoms related to stress, anxiety, and depression, scored on a four-point Likert scale from 0 (does not apply to me at all) to 3 (applies to me most of the time). Higher scores indicate higher psychological discomfort. The scores for each subscale are established by adding the scores of the individual items and multiplying the result by two. The DASS-21 scale, translated into Arabic, demonstrates strong psychometric properties [27].

The constructs of Depression, Anxiety, and Stress had an overall Cronbach’s alpha value of 0.851, 0.788 and 0.843, respectively, which implies that the items which measured Depression, Anxiety, and Stress were reliable [28]. Furthermore, it is noteworthy that none of the items in the column “Cronbach’s alpha if item deleted” had a value greater than the overall value of Cronbach’s alpha with respect to the corresponding construct; therefore, it seemed logical to keep all the items for further analysis. Moreover, it is apparent that all items have “item-rest correlations” above 0.20, signifying that the items which measure Depression, Anxiety, and Stress are consistent [29].

**Social Support Items**: Three self-developed questions assessed perceived support from family, friends, and faculty members. Participants responded using a frequency-based scale: Always, Frequently, Sometimes, Rarely, and Never.

### 2.5. Data Analysis

The demographic details of the participants were summarized in the form of frequency and percentages. Reliability assessment was done by employing Cronbach’s alpha coefficient to determine the reliability of items within the construct. Further, an evaluation of internal consistency was carried out using item rest correlation. Comparison of mean depression, anxiety, and stress scores between demographic and clinical groups was done by employing analysis of variance (ANOVA). Significant differences were considered at *p*-values below 0.05. All analyses were performed using JAMOVI software (version 2.3.28; The JAMOVI Project, Sydney, Australia). A post hoc sensitivity power analysis was performed utilizing. The study had 80% power to find a small-to-moderate effect size (f = 0.18) for group comparisons, with a significance level of α = 0.05 and a total sample size of 282. This shows that the achieved sample size was appropriate for the main inferential analyses.

## 3. Results

Table 1 shows the demographic characteristics of the participants. The study included 282 participants, most of whom were female, Kuwaiti, and unmarried. The majority were aged 18–22 years. Most participants were in their third year of study, followed by those in the second and fourth years. 

Table 2 summarizes the participants’ reported medical conditions and perceived social support. The majority reported not having any medical conditions, and among those with health issues, physical conditions were most common. Most described their conditions as mild, whereas fewer reported it as moderate and severe. With respect to social support, family and friends emerged as the primary sources of support, while faculty support was comparatively less prevalent. 

Table 3 reports the distribution of DASS-21 severity for Depression, Anxiety, and Stress. The findings indicate considerable variability in symptom severity across all three dimensions. For depression, participants were distributed across normal to extremely severe categories, indicating the presence of both low and elevated symptom levels within the sample. For anxiety, only a small fraction reported it as normal, whereas a considerable proportion reported extremely severe, moderate and severe anxiety. In contrast, stress scores were more evenly distributed across mild, moderate, severe, and extremely severe categories.

Table 4 shows the mean scores of depression, anxiety, and stress by demographic variables. The result shows that the female participants generally reported feeling more stressed compared to male participants, while depression and anxiety showed insignificant differences between male and female participants. 

The age groups did not show significant variation in depression, anxiety, and stress. People across different age, nationality and marital status groups reported similar depression, anxiety, and stress levels; i.e., the average scores of depression, anxiety, and stress levels were also not statistically significant different for different age, nationality and marital status groups.

However, year of study showed a different pattern. Second-year students generally had higher depression and stress scores compared to third- and fourth-year students. These differences were statistically significant, suggesting that students earlier in their studies might be facing more mental health challenges. However, the mean anxiety score did not show significant difference across different years of study.

Table 5 shows the mean depression, anxiety, and stress scores by health status and social support factors. People who said they had medical issues showed higher average depression and anxiety scores compared to those who did not have any. However, mean stress scores were quite similar regardless of health problems. When looking at types of conditions, those with mental health problems had higher mean depression, anxiety, and stress scores, but differences were not statistically significant. 

Further, the result showed that participants with severe conditions tended to feel more depressed, anxious, and stressed. However, the difference was statistically significant for stress level only. Having family support seemed to matter a lot. Those who rarely got support from family showed higher mean depression, anxiety, and stress scores. Support from friends followed a similar trend for anxiety, whereas participants who sometimes received support from friends reported higher depression level. The relationships for stress were weaker and not statistically significant. Support from faculty members was associated with depression and stress, with participants who always received faculty support experiencing lower symptom levels. 

## 4. Discussion

This study is, to our knowledge, the first to examine the prevalence and correlates of depression, anxiety, and stress among physiotherapy students in Kuwait. Our findings reveal a high burden of anxiety, with substantial proportions also reporting elevated depression and stress. These patterns align with international evidence that university students—particularly those in health professions—are vulnerable to psychological distress due to demanding academic and clinical requirements [16,30,31,32]. Our contribution adds context from Kuwait and highlights the role of social support as a potential protective factor within physiotherapy education.

### 4.1. Prevalence of Psychological Conditions

Mental health disorders among university students remain critically high worldwide. The Healthy Minds Study (2023/24) reported that 38% of students experienced moderate or severe depression and that 34% experienced moderate or severe anxiety [33,34]. These conditions are more prevalent in college students compared to age-matched peers [30,31], and similar trends are observed in the Arab region, emphasizing the need for mental health services and coping strategies [34]. Physiotherapy students appear particularly vulnerable compared to other disciplines, consistent with our findings of high stress, depression, and anxiety.

In our cohort, anxiety was the most prevalent condition, with nearly 40% reporting extremely severe levels. This finding aligns with global evidence that anxiety is widespread among university students [16] and is consistent with studies in the UAE and Malaysia [35,36]. Depression and stress were also common, though depression trailed anxiety—a gradient noted in Bangladeshi medical students [37]. Stress prevalence in our sample was lower than that reported among Saudi medical students (85.5%) [38], possibly reflecting discipline-specific demands. These observations underscore the urgent need for targeted interventions, as even reducing one stressor may not normalize overall distress [39].

### 4.2. Impact of Academic Year and Gender 

Our analyses revealed significantly higher depression and stress among second-year students compared to third- and fourth-year peers (*p* < 0.05), while anxiety did not differ by academic year. This suggests that early curricular transitions heighten vulnerability to low mood and strain, whereas anxiety remains relatively stable—a pattern supported by prior research [24,40]. Third-year students may benefit from clinical rotations, which provide structure and purpose, while fourth-year students face unique stressors such as burnout and career pressure [40]. Physiotherapy training combines rigorous academic coursework with physically demanding clinical rotations, which may contribute to elevated stress and anxiety. The need to master manual skills, manage patient expectations, and adapt to diverse clinical environments can exacerbate psychological strain [9,10]. Similar findings have been reported in Saudi cohorts [24].

The stability of anxiety across years aligns with studies showing that perceived stress decreases during placements while anxiety persists [41]. Nursing and health-profession students also report high anxiety during clinical exposure [32,42], and Doctor of Physical Therapy students exhibit elevated stress and anxiety without year-based differences [43]. These findings suggest that anxiety may require continuous attention throughout training.

Gender differences were evident: female students reported higher stress and tended toward higher anxiety, consistent with literature indicating greater psychological distress among women in higher education [25,26,44]. Given the gender imbalance in our sample, replication in more balanced cohorts is warranted. Prior work links workload and personal stressors to psychological symptoms in physiotherapy students [12,18], reinforcing the need for gender-sensitive and workload-aware interventions. 

### 4.3. The Effect of Social Support 

Social support emerged as a critical correlate of psychological well-being. Family support showed the strongest inverse association with depression, anxiety, and stress; faculty support was significantly linked to depression and stress but not anxiety (*p* = 0.043 and *p* = 0.032 vs. *p* = 0.113); and friends’ support was related primarily to depression and anxiety. Students who “rarely” reported family or faculty support exhibited the highest DASS-21 scores, while those who “always” felt supported had the lowest— a dose–response pattern, suggesting that meaningful relationships play an important role.

These findings align with evidence that higher perceived social support predicts lower psychological distress [4], though some cohorts (e.g., female students in Spain) report null associations [45]. Mechanistically, social support may buffer stress appraisal, enhance coping resources, and foster belonging—particularly salient in clinically intensive programs. Our factor-specific pattern suggests that family and peer engagement may be most relevant for anxiety, while faculty mentoring may influence mood and strain. Longitudinal studies are needed to confirm directionality and test whether strengthening support networks reduces distress [4,5,45,46].

## 5. Implications for Clinical Practice and Education

The significance of this study lies in its potential to advance knowledge in the field of student mental health. By providing detailed insights into the psychological challenges faced by physiotherapy students, this study contributes to the broader understanding of mental health in demanding academic fields. Furthermore, the findings can inform the development of targeted interventions and support systems within the College of Allied Health Sciences at Kuwait University, aimed at reducing stress and improving student well-being. 

Our findings have several practical implications: *Transition-sensitive support*: Implement early-year monitoring and coping workshops to address second-year vulnerability [24,40]. *Clinical-year scaffolding*: Provide structured debriefs and faculty mentoring during rotations, given links between faculty interaction and well-being [5,46]. *Gender-responsive strategies:* Tailor interventions to address higher stress among female students [25,26,44]. *Integrated support ecosystems*: Expand counseling access, promote wellness programs, and engage families and peers to strengthen protective networks [41]. *Curricular adjustments:* Calibrate workload during clinical blocks and offer flexible scheduling to reduce cumulative strain. These recommendations are hypothesis-generating; prospective studies should evaluate whether enhanced social support and workload calibration reduce DASS-21 scores across semesters [4,5,12,18,45,46]. Building on recent evidence from Kuwait University, which demonstrated the effectiveness of a stress management program in reducing stress and depression [22], our results suggest that combining these interventions with strategies to strengthen social support could further optimize mental health outcomes.

## 6. Limitations and Future Directions

This study has several limitations that should be acknowledged. First, the reliance on self-reported data may introduce response bias. Second, the cross-sectional design precludes any inference of causality; findings should be interpreted as associations rather than causal relationships. Third, the assessment of social support relied on three self-developed items, which may not capture its multidimensional nature. Future studies should employ validated instruments to enhance measurement accuracy. Fourth, the absence of a comparison group limits the ability to determine whether these findings are unique to physiotherapy students or generalizable to other health science or university students. Fifth, the gender imbalance, reflecting program demographics, may restrict generalizability across genders. Future research could explore gender-specific factors in more depth or include comparative analysis across institutions with balanced representation. Finally, longitudinal and qualitative studies are needed to track psychological well-being over time and provide deeper insights into students’ experiences. Intervention trials should test whether mentoring, advising, and family engagement reduce psychological distress and whether effects vary by academic year or gender. 

## 7. Conclusions

This study highlights the high prevalence of depression, anxiety, and stress among physiotherapy students in Kuwait and identifies key factors associated with these psychological outcomes, including gender, academic year, and perceived social support. Our findings emphasize the protective role of family and faculty support in mitigating psychological distress. These results underscore the need for further research to develop evidence-based interventions tailored to physiotherapy students’ unique academic and clinical challenges.

## Figures and Tables

**Table 1:** Demographic Characteristics.

	Overall (N = 282)	%
**Gender**		
Male	23	8.2%
Female	259	91.8%
**Age group**		
18–22	251	89.0%
23–27	29	10.3%
28–32	1	0.4%
33–37	1	0.4%
**Nationality**		
Kuwaiti	227	80.5%
Non-Kuwaiti	55	19.5%
**Marital Status**		
Single	269	95.4%
Married	12	4.3%
Divorced	1	0.4%
**Year of Study**		
2nd Year	90	31.9%
3rd Year	125	44.3%
4th Year	67	23.8%

**Table 2:** Medical conditions and perceived social support.

	Overall (N = 282)
**Do you have any medical conditions**	
Yes	59 (20.9%)
No	223 (79.1%)
**If yes, Type of the medical condition(s) is:**	
Mental	4 (6.8%)
Physical	43 (72.9%)
Both	12 (20.3%)
**Identify this severity of your conditions**	
Mild	35 (59.3%)
Moderate	21 (35.6%)
Severe	3 (5.1%)
**Do you receive family support**	
Never	6 (2.1%)
Rarely	12 (4.3%)
Sometimes	61 (21.6%)
Frequently	47 (16.7%)
Always	156 (55.3%)
**Do you receive support from your friends**	
Never	14 (5.0%)
Rarely	17 (6.0%)
Sometimes	75 (26.6%)
Frequently	70 (24.8%)
Always	106 (37.6%)
**Do you receive support from faculty members department:**	
Never	70 (24.8%)
Rarely	85 (30.1%)
Sometimes	85 (30.1%)
Frequently	24 (8.5%)
Always	18 (6.4%)

**Table 3:** Distribution of DASS-21 severity for Depression, Anxiety, and Stress.

	Overall (N = 282)
**Depression**	
Normal	102 (36.2%)
Mild	38 (13.5%)
Moderate	71 (25.2%)
Severe	31 (11.0%)
Extremely Severe	40 (14.2%)
**Anxiety**	
Normal	45 (16.0%)
Mild	15 (5.3%)
Moderate	66 (23.4%)
Severe	45 (16.0%)
Extremely Severe	111 (39.4%)
**Stress**	
Normal	103 (36.5%)
Mild	43 (15.2%)
Moderate	50 (17.7%)
Severe	54 (19.1%)
Extremely Severe	32 (11.3%)

**Table 4:** Mean scores of depression, anxiety, and stress by demographic variables.

Variable		Depression	*p*	Anxiety	*p*	Stress	*p*
		Mean ± SD		Mean ± SD		Mean ± SD	
Gender	Male	12.5 ± 10.7	0.335	13.4 ± 11.8	0.057	14.3 ± 10.7	0.015
	Female	14.8 ± 10.6		17.5 ± 9.7		19.8 ± 10.2	
Age-group	18–22	14.5 ± 10.2	0.667	17.4 ± 9.7	0.426	19.4 ± 10.4	0.893
	23–27	15.7 ± 14.1		16.2 ± 11.6		19.5 ± 10.6	
	28–32	20.0 ± 0.0		14.0 ± 0.0		16.0 ± 0.0	
	33–37	4.0 ± 0.0		2.0 ± 0.0		12.0 ± 0.0	
Nationality	Kuwaiti	14.1 ± 10.4	0.119	17.1 ± 9.8	0.750	19.4 ± 10.4	0.957
	Non- Kuwaiti	16.6 ± 11.2		17.6 ± 10.4		19.3 ± 10.3	
Marital Status	Single	14.6 ± 10.7	0.706	17.3 ± 9.9	0.501	19.4 ± 10.4	0.851
	Married	13.2 ± 9.1		14.0 ± 10.9		18.7 ± 9.5	
	Divorced	22.0 ± 0.0		14.0 ± 0.0		14.0 ± 0.0	
Year of Study	2nd Year	16.3 ± 11.1	0.014	18.5 ± 9.2	0.293	20.8 ± 10.5	0.043
	3rd Year	12.5 ± 8.5		16.4 ± 8.8		17.6 ± 9.5	
	4th Year	16.1 ± 12.8		16.7 ± 12.4		20.6 ± 11.3	

**Table 5:** Mean depression, anxiety, and stress scores by health status and social support factors.

Variable		Depression	*p*	Anxiety	*p*	Stress	*p*
		Mean ± SD		Mean ± SD		Mean ± SD	
Do you have medical conditions	Yes	19.3 ± 11.7	<0.001	20.7 ± 11.7	0.007	20.2 ± 9.7	0.530
	No	13.7 ± 10.2		16.5 ± 9.4		19.2 ± 10.5	
Type of medical condition	Mental	27.0 ± 13.2	0.060	26.0 ± 9.4	0.271	26.5 ± 4.4	0.090
	Physical	16.2 ± 10.6		18.5 ± 11.3		18.4 ± 9.5	
	Both	22.3 ± 10.6		22.8 ± 11.6		23.7 ± 9.5	
Severity of your condition	Mild	16.9 ± 11.4	0.124	17.5 ± 11.4	0.061	17.1 ± 9.5	0.005
	Moderate	17.8 ± 10.0		21.2 ± 9.6		22.6 ± 7.7	
	Severe	30.7 ± 13.3		32.7 ± 16.2		32.7 ± 11.4	
Receive family support	Never	7.3 ± 8.3	<0.001	4.7 ± 7.3	<0.001	9.0 ± 6.7	<0.001
	Rarely	25.0 ± 12.0		23.8 ± 10.3		28.5 ± 6.3	
	Sometimes	18.0 ± 10.7		18.9 ± 9.8		21.9 ± 9.6	
	Frequently	17.9 ± 10.3		18.3 ± 9.9		19.9 ± 9.8	
	Always	11.7 ± 9.5		16.1 ± 9.5		17.8 ± 10.5	
Receive support from your friends	Never	11.6 ± 9.4	<0.001	12.4 ± 8.9	0.033	15.6 ± 10.1	0.154
	Rarely	15.2 ± 11.6		20.4 ± 11.2		20.2 ± 10.9	
	Sometimes	17.8 ± 10.9		18.8 ± 10.0		20.7 ± 9.8	
	Frequently	16.5 ± 10.5		18.1 ± 8.6		20.7 ± 10.5	
	Always	11.3 ± 9.6		15.5 ± 10.3		17.8 ± 10.4	
Receive support from faculty members department	Never	15.7 ± 10.4	0.043	17.9 ± 10.3	0.113	19.7 ± 10.9	0.032
	Rarely	16.6 ± 10.2		19.0 ± 9.8		21.9 ± 9.5	
	Sometimes	13.5 ± 11.3		16.0 ± 9.8		17.2 ± 10.0	
	Frequently	11.3 ± 8.8		14.8 ± 9.1		19.0 ± 10.4	
	Always	10.2 ± 10.3		14.0 ± 10.0		16.6 ± 11.5	

## Data Availability

The data that support the findings of this study are not openly available due to reasons of sensitivity and are available from the corresponding author upon reasonable request.
